# Radiographic and patient-specific predictors of poor outcome following hip reconstruction in children with cerebral palsy

**DOI:** 10.1186/s12887-026-06910-7

**Published:** 2026-04-28

**Authors:** Stefanos Tsitlakidis, Angelika Kolmann, Paul Mick, Johannes Weishorn, Julius Stupp, Pit Hetto, Nicholas A. Beckmann

**Affiliations:** Clinic for Orthopedics, Heidelberg University Hospital, Schlierbacher Landstr. 200a, Heidelberg, Baden- Wuerttemberg 69118 Germany

**Keywords:** Cerebral palsy, Hip dysplasia/(sub)luxation, Varus osteotomy/Dega acetabuloplasty, FEAR index, Radiographic outcome

## Abstract

**Aims:**

Patients with spastic cerebral palsy (CP) are at a high risk of neurogenic hip dysplasia/subluxation depending on the severity of the neuromuscular disorder. Untreated, approximately one third of all patients develop hip dislocation. Reconstruction with femoral varus derotational osteotomy (VDRO) combined with Dega acetabuloplasty (PO) represents the gold standard. The goal of this study was the radiographic assessment after reconstructive treatment of spastic hip dysplasia/(sub)luxation and to derive specific thresholds and target values of neck shaft angle NSA) and femoro-epiphyseal acetabular roof (FEAR)-index that could be beneficial in predicting long-term outcome.

**Methods:**

In this retrospective evaluation, 121 patients (224 hips) with CP who underwent VDRO/acetabuloplasty were grouped according to their age at surgery and postoperative radiographic parameters (NSA and FEAR-index) and compared with each other over time (5-year follow-up). The preoperative, postoperative and follow-up X-rays were analyzed. For this purpose, the FEAR, lateral center-edge angle (LCE) and migration percentage (MP) were analyzed as outcome measures at hip-level using linear mixed models (LMM).

**Results:**

Patients older than 8 years and with a postoperative FEAR > -20° or a postoperative NSA > 130° showed a significantly worse postoperative result (FEAR, LCE and MP). A deterioration of the outcome parameters was found in all subgroups to approximately the same extent up to 2 years postoperatively. After 5 years, the findings remained stable. Failure rates and relative risks of inferior subgroups (FEAR-index ≥-20°, NSA ≥ 130°, age ≥ 8 years) were approximately twice as high (n_AGE_ 18/114 vs. 34/110; n_FEAR_ 20/150 vs. 25/74; n_NSA_ 22/132 vs. 27/92).

**Conclusion:**

A sufficient postoperative head coverage/reduction of MP and thus joint stability is crucial for long-term outcomes after VDRO and PO. Particularly the FEAR-index seems to be a useful parameters for the surgeon for preoperative planning and postoperative aftercare. If postoperative risk factors are present, an individualized aftercare program and hip monitoring plan that establishes more frequent postoperative assessment and possible prolonged abduction therapy should be considered.

## Article summary

### Article focus

- Assessment of patient-specific and radiographic parameters for the outcome after hip reconstruction.
- Surgeons should opt for these as target values.

### Key messages

- Age <8 years, a postoperative FEAR-index of <-20° and a postoperative NSA of <130° seem to be beneficial for long-term outcomes
- Surgeons should opt for these as target values.
- Hips with FEAR ≥-20°, NSA ≥130° or age ≥8 years at surgery had roughly 2× higher risk of failure. Patients fulfilling all three “highrisk” criteria had a ~3-fold higher failure risk.

### Strengths and limitations

- Large patient cohort with 5-year follow-up.
- First study to use the FEAR-index in this context.
- Retrospective study design.

## Introduction

Cerebral palsy (CP) is a heterogeneous and complex neuromuscular disorder and is associated with varying degrees of severity [1–4]. Neurogenic hip dysplasia/dislocation represents an often encountered and relevant condition in these patients caused by spasticity and leading to pain and increased impairment that negatively affects the health-related quality of life [5–8]. Hip surveillance programs that assess hip displacement to guide decision making have been proven effective in reducing the risk and frequency of dislocated hips, pain, and impairment thus improving health-related quality of life [5, 7, 8]. In case of severe neurogenic spastic hip dysplasia and dislocation, femoral varus derotational osteotomy (VDRO), isolated or combined with pelvic osteotomy (PO) represents an established procedure for reconstructive treatment [9–12]. In this context, a combination of femoral and pelvic osteotomy is the most effective approach especially for severe spastic hip dysplasia and is generally considered beneficial for outcomes and long-term results [10–15]. To date, there is strong evidence that the migration percentage (MP) is the main and most important parameter, as it is reproducible with excellent intrarater and interrater reliability [6, 16–18].

However, it remains unclear, if and which directly surgically modifiable parameters can be used for outcome parameters and which target values should be aimed for. In this context, surgically modifiable radiographic parameters, such as the femoro-epiphyseal acetabular roof (FEAR)-index [19] and the neck shaft angle (NSA), may have an impact on hip survival and thus the outcome after reconstruction surgery. Once specified, these surgically modifiable parameters could be used as predictors for the outcome after surgical treatment.

Therefore, the goal of this study was the radiographic assessment after reconstructive treatment of spastic hip dysplasia/(sub)luxation and to derive specific thresholds and target values of FEAR-index and NSA that could be beneficial in predicting long-term outcome. These values could also assist in the preoperative planning of reconstruction surgery.

## Patients & methods

This study was conducted as a single center retrospective cohort study after approval by the local ethics committee (S-198_2019/2024). In adherence with the ethics committee, an additional/specific consent to participate of parents or legal guardians for minors (younger than the age of 16) was not necessary, as this work was conducted as a retrospective analysis.

### Study population

A total of 121 skeletally immature patients (224 hips) were included in this retrospective mono-centric radiographic follow-up assessment. Reconstruction surgery was performed between 06/2010 and 05/2020.

The inclusion criteria for the patients were:

- patients with CP (bilateral and unilateral).
- neurogenic/spastic hip dysplasia/subluxation (including dislocation) with a migration percentage (MP) of > 40%.
- no previous surgery (in terms of femoral or pelvic osteotomies).

Subgroups were established after receiver operating characteristic (ROC)-curve analysis based on patient age at the time of surgery, the *postoperative* FEAR-index [19] and *postoperative* NSA.

### Surgical procedure and aftercare

**Fig. 1:**
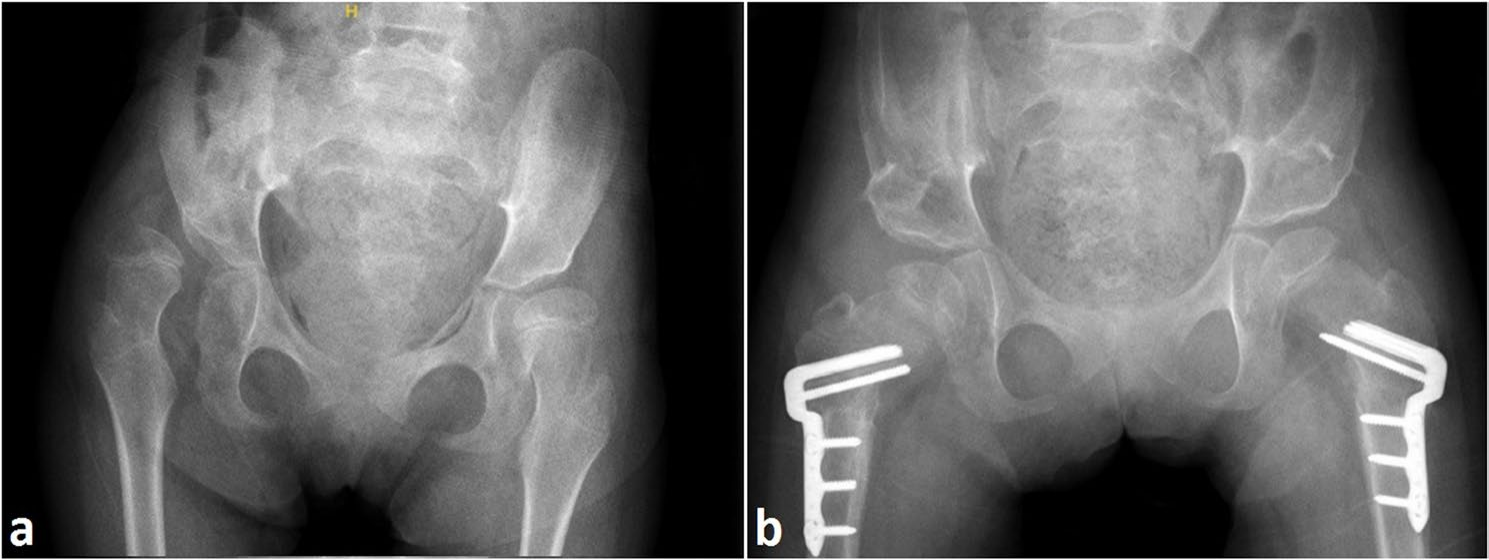
Pelvic radiographs. (**a**) showing the preoperative condition: dislocated right and subluxated left hip; (**b**) postoperative outcome at the 2-year follow up: VDRO and Dega osteotomy on both sides with reduced and stable hips

Once the indication for hip reconstructive treatment was made/put (preoperative MP > 40%), patients were scheduled for surgery. The surgical procedure included (when necessary/for dislocated hips) an open reduction through an anterior approach to the hip and open tenotomies of the adductor longus/gracilis and the iliopsoas, combined with a simultaneous femoral varus derotational osteotomy (VDRO) through a lateral approach and Dega´s acetabuloplasty (PO) (Fig. 1). The amount of varization/derotation was determined individually to achieve sufficient reduction of the femoral head/head coverage. For internal fixation of the VDRO the 3.5 PediLoc^®^ Locking Cannulated Blade Plate (Orthopediatrics, Warsaw, IN, USA) was used. For stabilization of Dega acetabuloplasty the autograft osseous wedges taken during the concurrent femoral shortening osteotomy were used.

Postoperative aftercare included regular physical therapy, a gradual increase in range of motion (ROM), and a gradual increase in weight-bearing after 4 weeks of non-weight bearing and the use of an abduction foam system for abduction at night for 12 months after the surgery. Non-ambulatory patients also had abduction padding integrated to their wheelchairs.

### Radiographic assessment/follow-up

Standard supine anteroposterior pelvic radiographs were analyzed preoperatively (preop.), immediately postoperatively (T0), after one year (T1), at the 2-year follow-up (T2, Fig. 1) and at the 5-year follow-up (T5). The migration percentage (MP), lateral center edge angle (LCE) and FEAR-index were used and analyzed as outcome measures. Failure was defined as subsequent MP greater than 40%, as it indicates substantial migration that is considered to need surgical reconstruction [20–23].

### Statistics

Data was structured using Microsoft Excel (Microsoft, Redmond, WA, USA) and analyzed using SPSS Version 25.0 (IBM, Chicago, IL, USA).

Multivariable linear regression analysis was performed with MP at final follow-up as the outcome and age at surgery, postop. FEAR-index and postop. NSA as covariates to assess the influence of these factors.

A receiver operating characteristic (ROC) curve analysis was performed in order to determine cutoff values for age at surgery (as a *preoperative* parameter) as well as postoperative FEAR-index and postoperative NSA at final follow-up to distinguish between satisfactory and poor outcome (MP > 40%) in advance of subgroup allocation and analyses. The values with the highest sensitivity and specificity are represented by the infliction point of the ROC curves and indicate a valuable cut-off value for the different factors. Additionally, an area-under-the-curve (AUC) analysis was performed, in order to assess for statistical significance.

For additional correlation analysis of the Pearson product moment correlation coefficient (PPMC) was calculated.

For descriptive statistics the arithmetic mean and standard deviation were calculated. The subsequent comparative statistics of continuous variables between the subgroups was performed using linear mixed models to account for bilaterality. Mean differences with corresponding 95% confidence intervals (CI) were included as absolute estimates of (fixed) effects.

X² test was performed for the comparison of categorical variables (failure rates). Relative Risks were calculated using binomial (Crosstabs) model.

The level of significance was set at *p* < 0.05 for all performed analyses.

## Results

### Study population/patient characteristics

In total, 121 individuals (59 females, 62 males, 224 hips) met our inclusion criteria and were included in this study. Of the 121 patients, 103 patients were affected and treated bilaterally (hips), 18 patients unilaterally.

The demographic characteristics of the total cohort are displayed in Table 1.

**Table 1:** Demographic baseline characteristics of the total study population (patient-level & hip-level)

variable	total cohort
number of patients	121
number of hips	224
m: f ratio [patients]	62:59
m: f ratio [hips]	114:110
patient´s age at surgery [years ± SD]	7.9 ± 3.6
GMFCS level distribution, n pat (%)	
I	9 (7.5%)
II	20 (16.5%)
III	14 (11.6%)
IV	30 (25%)
V	48 (39.3%)

### Multivariable, ROC and correlation analyses

The results of the multivariable regression analysis are given in Table 2. Age at surgery, the FEAR-index and NSA significantly influenced the risk for failure, whereas for the GMFCS levels, the results indicate no influence on the outcome.

**Table 2:** Results of multivariate (linear regression) analysis

variable	multivariate	
	coefficient (95% CI)	*p*-value
age at surgery	-0.533 (-1.038 – -0.28)	**0.039**
GMFCS level	0.747 (-0.674–2.168)	0.301
postop. FEAR-index	0.252 (0.129–0.376)	**< 0.001**
postop. NSA	-0.147 (-0.320–0.025)	**0.049**

Significant values are presented in bold letters

**Fig. 2:**
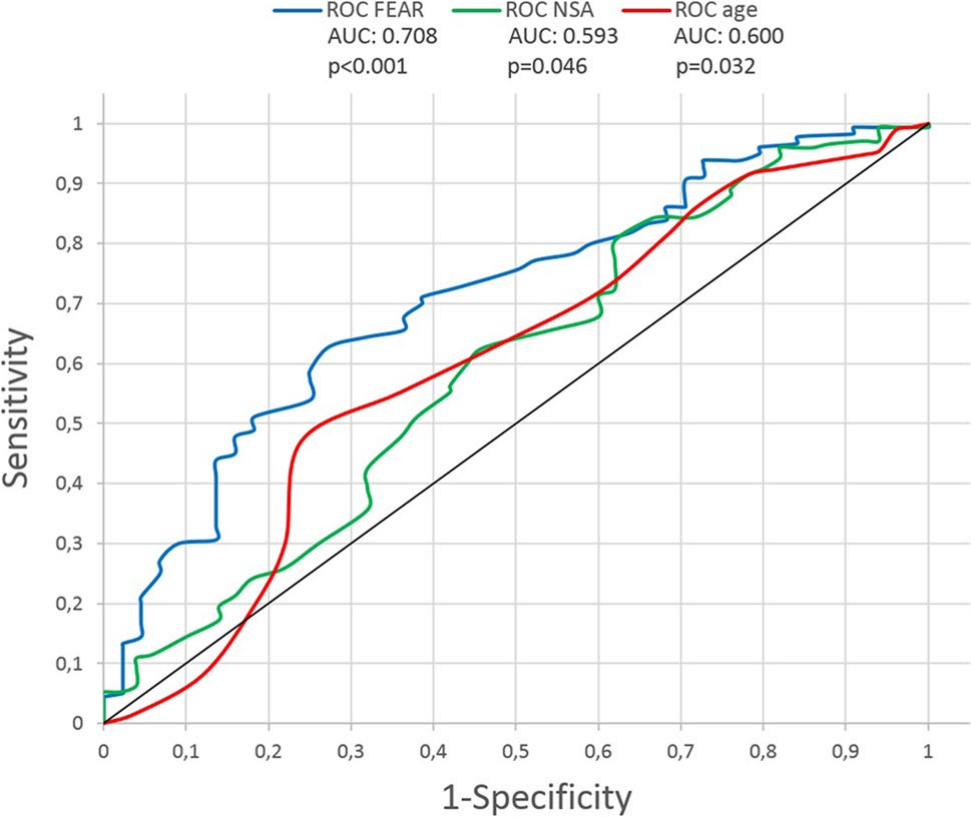
ROC-curve analysis

The ROC-curve analysis included age at surgery as a preoperative parameter as well as postoperative FEAR-index and postoperative NSA (Fig. 2).

Age at surgery of 7.5 years was identified to be the threshold for a satisfactory outcome (63% sensitivity; 52% specificity; AUC = 0.600, 95% CI = 0.506–0.694; *p* = 0.032). A FEAR-index of -20,5° was identified to be the threshold for a satisfactory outcome (71% sensitivity; 62% specificity; AUC = 0.708, 95% CI = 0.625–0.792; *p* < 0.001). A NSA of 129.5° was identified to be the threshold for a satisfactory outcome (63% sensitivity; 54% specificity; AUC = 0.593, 95% CI = 0.499–0.686; *p* = 0.046).

For further analysis subgroups were defined for age as <8y (7 years or younger) and ≥ 8y (8 years or older). For the subgroup analysis with regard to radiographic parameters, subgroups were defined as NSA < 130° (129° or lower) and NSA ≥ 130° (130° or higher) and for the FEAR-index as FEAR <-20° (-21° or lower) and FEAR ≥ -20 (-20° or higher). All subgroup defining variables were measured/recorded without decimals. Furthermore, particularly for the NSA and FEAR-index, a surgical correction as precise as 0.5° is not reasonably achievable.

The results of the correlation analysis are given in Table 3. While the FEAR-index and NSA showed very strong and moderate correlations respectively, for both parameters correlations decreased over time.

**Table 3:** Results of the the correlation analysis

**Variables**		MP T0	MP T1	MP T2	MP T5
postop. FEAR-index	coefficient	0.75	0.45	0.33	0.20
	*p-value*	**< 0.0001**	**< 0.0001**	**< 0.0001**	**0.004**
postop. NSA	coefficient	0.45	0.21	0.10	0.00
	*p-value*	**< 0.0001**	**0.003**	*0.143*	*0.998*

Significant values are presented in bold letters

### Radiographic assessment

The results (mean ± SD) of the preoperatively and postoperatively assessed outcome measures and the corresponding p-values applying linear mixed models to account for bilaterality are given in Tables 4, 5 and 6. The analyzed preoperative coxometric parameters (FEAR-index, LCE, MP) showed no statistically significant differences between the subgroups (Tables 4, 5 and 6).

**Table 4:** Outcome measures (mean ± SD) per time point including corresponding *p*-values (linear mixed effect model) by patient-specific characteristics

		total (mean ± SD)	age < 8y (mean ± SD)	age ≥ 8 y (mean ± SD)	estimates of effect mean difference (95% CI)	*p*-values
*n* (hips)		224	114	110		
age (mean ± SD years)		7.9 ± 3.6	5.0 ± 1.4	10.9 ± 2.6		**< 0.001**
PREOP.	**FEAR**	21.4 ± 18.1	21.3 ± 16.9	21.4 ± 19.4	0.6 (-4.6–5.8)	0.826
	**LCE**	-3.7 ± 18.1	-4.7 ± 17.7	-2.6 ± 18.5	-2.1 (-6.8–2.7)	0.386
	**MP**	48.3 ± 23.8	48.1 ± 25.3	48.6 ± 22.2	1.8 (-3.2–6.8)	0.470
T0	**FEAR**	-27.9 ± 19.8	-32.1 ± 18.8	-23.7 ± 20.1	-8.4 (-13.9 – -2.9)	**0.003**
	**LCE**	11.4 ± 7.8	11.1 ± 6.8	11.8 ± 8.9	-0.8 (-2.9–1.4)	0.480
	**MP**	14.5 ± 16.2	10.3 ± 13.7	18.7 ± 17.6	-8.3 (-12.8 – -3.7)	**< 0.001**
T1	**FEAR**	-13.7 ± 17.9	-14.0 ± 17.3	-13.4 ± 18.6	-0.8 (-6.1–4.4)	0.756
	**LCE**	12.8 ± 9.0	12.4 ± 7.7	13.3 ± 10.3	-0.9 (-3.5–1.7)	0.501
	**MP**	23.1 ± 16.2	20.8 ± 16.1	25.7 ± 16.0	-5.0 (-9.8 – -0.1)	**0.044**
T2	**FEAR**	-10.1 ± 15.9	-9.3 ± 13.5	-11.2 ± 18.4	1.9 (-2.9–6.8)	0.439
	**LCE**	12.3 ± 8.5	11.4 ± 8.2	13.4 ± 8.7	-2.0 (-4.7–0.7)	0.146
	**MP**	29.9 ± 13.7	28.4 ± 13.0	32.0 ± 14.6	-3.2 (-7.7–1.3)	0.164
T5	**FEAR**	-10.1 ± 16.1	-7.3 ± 14.5	-13.5 ± 17.3	6.3 (1.6–10.7)	**0.008**
	**LCE**	16.4 ± 10.6	14.4 ± 9.7	18.6 ± 11.0	-4.4 (-7.4 – -1.4)	**0.005**
	**MP**	28.1 ± 12.8	28.1 ± 12.4	27.9 ± 13.3	0.7 (-3.2–4.6)	0.735

Significant values are presented in bold letters

**Table 5:** Outcome measures (mean ± SD) per time point including corresponding *p*-values (linear mixed effect model) by postop. FEAR-index

		postop. FEAR < -20° (mean ± SD)	postop. FEAR ≥ -20° (mean ± SD)	estimates of effect mean differences (95% CI)	*p*-values
*n* (hips)		150	74		
age (mean ± SD years)		8.0 ± 2.7	7.8 ± 3.8		0.529
PREOP.	**FEAR**	21.7 ± 18.8	20.7 ± 16.7	-0.8 (-5.9–4.2)	0.753
	**LCE**	-4.5 ± 18.7	-2.0 ± 16.6	1.8 (-3.3–6.8)	0.496
	**MP**	52.1 ± 18.8	51.0 ± 19.5	-0.4 (-5.7–4.9)	0.888
T0	**FEAR**	-38.6 ± 12.2	-6.2 ± 13.4	32.5 (28.9–36.0)	**< 0.001**
	**LCE**	12.7 ± 7.0	8.7 ± 8.5	-4.3 (-6.4 – -2.2)	**< 0.001**
	**MP**	6.9 ± 11.4	29.7 ± 13.7	22.1 (18.7–25.5)	**< 0.001**
T1	**FEAR**	-19.6 ± 14.2	-2.2 ± 18.8.	17.7 (12.9–22.4)	**< 0.001**
	**LCE**	14.3 ± 6.8	9.7 ± 11.8	-5.1 (-7.7 – -2.6)	**< 0.001**
	**MP**	18.7 ± 13.4	32.2 ± 17.7	13.5 (9.1–17.8)	**< 0.001**
T2	**FEAR**	-14.0 ± 15.1	-2.5 ± 14.6	11.6 (6.9–16.3)	**< 0.001**
	**LCE**	13.4 ± 8.0	10.2 ± 9.1	-3.3 (-5.8 – -0.7)	**0.012**
	**MP**	27.0 ± 13.1	35.2 ± 13.3	7.2 (3.2–11.3)	**0.001**
T5	**FEAR**	-11.7 ± 16.0	-6.2 ± 15.6	5.9 (1.2–10.7)	**0.014**
	**LCE**	17.0 ± 9.9	15.3 ± 11.9	-1.9 (-4.8–1.0)	0.189
	**MP**	26.8 ± 12.4	30.4 ± 13.4	3.2 (-0.2–6.7)	**0.049**

Significant values are presented in bold letters

**Table 6:** Outcome measures (mean ± SD) per time point including corresponding *p*-values (linear mixed effect model) by postop. NSA

		postop. NSA < 130° (mean ± SD)	postop. NSA ≥ 130° (mean ± SD)	estimates of effect mean difference (95% CI)	*p*-values
*n* (hips)		132	92		
age (mean ± SD years)		8.1 ± 2.9	7.9 3.7		0.808
PREOP.	**FEAR**	22.8 ± 19.3	19.4 ± 16.2	-3.7 (-8.6–1.2)	0.134
	**LCE**	-7.2 ± 19.9	-1.4 ± 13.8	8.5 (3.8–13.2)	**< 0.001**
	**MP**	53.7 ± 19.5	48.9 ± 17.9	-4.7 (-9.8–0.3)	0.066
T0	**FEAR**	-36.0 ± 16.3	-16.3 ± 18.7	19.5 (14.8–24.2)	**< 0.001**
	**LCE**	11.9 ± 7.0	10.7 ± 8.7	-1.4 (-3.5–0.7)	0.195
	**MP**	9.2 ± 12.5	21.9 ± 18.0	12.3 (8.2–16.3)	**< 0.001**
T1	**FEAR**	-18.3 ± 16.1	-7.2 ± 18.4	11.1 (6.3–16.0)	**< 0.001**
	**LCE**	13.3 ± 7.0	12.0 ± 11.4	1.3 (-3.8–1.3)	0.325
	**MP**	20.5 ± 14.0	26.9 ± 18.4	6.2 (1.6–10.7)	**0.008**
T2	**FEAR**	-12.3 ± 15.9	-7.0 ± 15.3	5.1 (0.4–9.7)	**0.034**
	**LCE**	11.9 ± 8.7	12.8 ± 8.1	0.7 (-1.8–3.2)	0.573
	**MP**	28.7 ± 14.1	32.0 ± 13.2	2.6 (-1.4–6.7)	0.204
T5	**FEAR**	-11.2 ± 16.9	-8.7 ± 14.9	2.2 (-2.4–6.7)	0.355
	**LCE**	15.7 ± 11.2	17.6 ± 9.5	1.4 (-1.4–4.3)	0.329
	**MP**	27.8 ± 13.7	28.3 ± 11.5	0.6 (-2.8–4 − 0)	0.745

Significant values are presented in bold letters

There was no statistically significant difference in the preoperative radiographic parameters and particularly regarding the MP values between the different subgroups (age, FEAR, NSA).

Significant improvements after VDRO + PO in all outcome measures were evident for all different subgroups (Tables 4, 5 and 6).

The study cohort in total and all subgroups showed significant improvements in all radiographic parameters immediately postoperatively (Tables 4, 5 and 6, T0). A deterioration (as indicated by an increase of the MP) was evident for all subgroups until the 2-year follow-up (T2). Between the 2-year and 5-year follow-up assessments results remained stable (Tables 4, 5 and 6, T2/T5).

Throughout the observation period the subgroups with an age < 8 years, a FEAR-index <-20° and a NSA < 130° showed better outcomes with regard to the analyzed radiographic parameters. The FEAR <-20° subgroup in particular, showed significantly better outcomes in all of the radiographic measures throughout almost the entire observation period (Table 5).

These subgroups (FEAR-index <-20°, NSA < 130°, age < 8 years) showed a failure rate of 13–17%. The overall failure rate was 22%. The failure rates and relative risks of failure of the other subgroups (FEAR-index ≥-20°, NSA ≥ 130°, age ≥ 8 years) was approximately twice as high (Table 7). There was no significant difference concerning the relative risks between the different GMFCS levels (with level I as the reference). Interestingly, the lowest failure rate was found in level II patients. Patients fulfilling all three “high-risk” criteria had a approximately threefold higher risk of failure (Table 7).

**Table 7:** Predictors of postoperative failure (MP > 40%) at final follow-up (hip-level)

Variable	Subgroup	Failures [*n*/*N*]	Failure rate [%]	Relative Risk (95% CI)	*p*-value
age at surgery	< 8 years	18 / 114	15.8	reference	**0.007**
	≥ 8 years	34 / 110	30.9	1.96 (1.2–3.3)	—
GMFCS	level I	3 / 17	17.6	reference	—
	level II	3 / 37	8.1	0.46 (0.10–2.05)	0.300
	level III	4 / 26	15.4	0.87 (0.22–3.42)	0.844
	level IV	10 / 56	17.9	1.01 (0.31–3.26)	0.984
	level V	25 / 88	28.4	1.61 (0.55–4.74)	0.358
postop. FEAR-index	< − 20°	20 / 150	13.3	reference	**< 0.001**
	≥ − 20°	25 / 74	33.8	2.54 (1.55–4.23)	—
postop. NSA	< 130°	22 / 132	16.7	reference	**0.034**
	≥ 130°	27 / 92	29.3	1.70 (0.76–2.10)	—
combined `high-risk` *(≥ 8y+* *FEAR≥-20°+* *NSA ≥ 130°)*	yes	15 / 38	39.5	2.8(1.6-5.0)	**< 0.001**
	no	37 / 186	19.9	reference	—

Significant values are presented in bold letters

## Discussion

Patients with CP are at high risk of neurogenic hip dysplasia/subluxation depending on the severity of the neuromuscular disorder. In this context, VDRO combined with a pelvic osteotomy represents an established procedure for reconstructive surgery of neurogenic spastic hip dysplasia and dislocation [9–12, 15].

The goal of this current study was a radiographic assessment after reconstructive surgery (VDRO and Dega´s acetabuloplasty) of spastic hip dysplasia/dislocation and to identify specific thresholds and target values of the FEAR-index and the NSA that should be aimed for during reconstructive surgery. To the best of our knowledge, this is the first study to evaluate the outcome after hip reconstruction surgery based on the surgically modifiable and postoperative FEAR-index and postoperative NSA.

Our results suggest that directly surgically modifiable parameters (particularly the FEAR-index ) and age at the time of surgery (patient-specific risk factor) have significant impact on long-term outcome after reconstruction surgery of subluxated/dislocated hips in CP (Tables 4, 5 and 6). All subgroups showed significant improvements in all analyzed radiographic parameters, particularly the MP measured immediately postoperatively (T0). A deterioration, as indicated by an increase of the MP value, was evident for all subgroups until the 2-year follow-up (T2). Afterwards, results remained stable (T5). Patients showing all three “high-risk” criteria were found to have a threefold higher risk of failure (Table 7).

Our findings are comparable to and are supported by those of other authors. Kiapekos et al. and Park et al. reported on their findings of proximal femoral osteotomies and combined femoral-pelvic osteotomies in children with CP [10, 11, 23, 24]. During their analysis, they found a slow increase of the MP postoperatively during the first three years [11, 23, 24]. From then on, until the 5-year follow-up the MP decreased [11]. The authors concluded that a lower postoperative MP contributed to a lower failure rate [10, 11]. The postoperative MP has been reported to be more important for the outcome than the preoperative MP [10, 22, 24].

Our reported failure rates during this work are comparable to those of prior studies and within the reported range [10, 11, 22, 24, 25].

With regard to age at surgery as a patient-specific risk factor, there are inconsistent results in prior studies [13, 21, 24, 26, 27]. As described, our results suggest an age > 8 years to be a risk factor for increased failure. However, an age < 6 years might also be unfavorable as over time deterioration (rebound coxa valga) will occur until skeletal maturity [5, 16]. Therefore, in this particular patient subpopulation, a combined femoral-pelvic osteotomy is recommended [9]. An age > 8 years is also considered unfavorable, as the time period for acetabular and femoral remodeling to occur prior to skeletal maturity is limited and likely insufficient [5, 28].

The NSA and FEAR-index are directly surgically modifiable parameters. Prior reports have analyzed the NSA and alternatively the head shaft angle (HSA) [29–31]. Both, the NSA and HSA, were reported to be significant predictors for postoperative improved acetabular remodeling and to be significant factors affecting the MP with higher values leading to unfavorable outcomes [29–31]. In contrast to the FEAR-index, the HSA defines the orientation of the proximal femoral physis relative to the shaft rather than the acetabular roof, to quantify the valgus of the femoral head. The HSA could therefore be utilized as an alternative to the FEAR-index as a measure of joint (in)stability.

So far, to the best of our knowledge, there are no studies reporting on and analyzing the influence of the FEAR-index on the outcome after hip reconstruction surgery in CP. A FEAR-index <-20°, indicating superior joint stability is associated with a more favorable outcome, as this subgroup showed better postoperative MP results throughout the whole observation period (Table 5). A postoperative MP as low as 5.1% has been reported as a threshold value associated with satisfactory outcomes in the long-term [10]. In our analysis, the subgroup with a postoperative FEAR-index <-20° showed an immediate postoperative MP of 6.9% (close to a previously reported MP value of 5.1% [10] and showed the best results among all subgroups (MP and failure rate, Tables 5 and 7).

Thus, this radiographic and patient-specific aspects indicate that surgeons should plan and opt for a sufficient varization preoperatively that leads and provides sufficient head coverage/reduction of MP and joint stability after VDRO + PO. In this context, the NSA and FEAR-index seem to be useful parameters. In the presence of postoperative risk factors,, the aftercare protocol should be individualized. Postoperative hip monitoring may include more frequent radiographic controls and suggest prolonged abduction therapy.

There are limitations to our study. Since our study primarily is based on radiographic findings, data on patient compliance concerning the aftercare protocol and particularly the postoperative abduction therapy over a 12-month period. However, patient’s compliance may influence on final outcomes and radiographic findings, thereby negatively affecting the interpretation of our results. Additionally, “failure” is exclusively defined based on radiographic findings (MP > 40%). Clinical endpoints, such as pain or functional outcomes are missing, again influencing the interpretation of our results.

## Conclusion

A sufficient postoperative head coverage/reduction of MP and thus joint stability is crucial for long-term outcomes after VDRO and PO. Particularly the FEAR-index seems to be useful parameters for the surgeon for preoperative planning and postoperative aftercare. If postoperative risk factors are present, an individualized aftercare plan and hip monitoring regime with more frequent postoperative controls with possible prolonged abduction therapy should be considered.

## Data Availability

The datasets supporting the conclusions of this article are included within the article.

## Abbreviations

AUC: Area under the curve
CI: Confidence interval
CP: Cerebral Palsy
FEAR: Femoro-epiphyseal acetabular roof
HSA: Head shaft angle
LCE: Lateral center edge angle
LMM: Linear mixed effect models
MP: Migration percentage
NSA: Neck shaft angle
PO: Dega acetabuloplasty / pelvic osteotomy
ROC: Receiver operating characteristic
ROM: Range of motion
VDRO: Varus derotational osteotomy
